# Phase-sensitive interferometry of decorrelated speckle patterns

**DOI:** 10.1038/s41598-019-47979-8

**Published:** 2019-08-13

**Authors:** Hendrik Spahr, Clara Pfäffle, Sazan Burhan, Lisa Kutzner, Felix Hilge, Gereon Hüttmann, Dierck Hillmann

**Affiliations:** 1 Institute of Biomedical Optics, University of Lübeck, Peter-Monnik-Weg 4, 23562 Lübeck, Germany; 2 Medical Laser Centre Lübeck GmbH, Peter-Monnik-Weg 4, 23562 Lübeck, Germany; 3 Airway Research Center North (ARCN), Member of the German Center for Lung Research (DZL), 22927 Großhansdorf, Germany; 4 Thorlabs GmbH, Maria-Goeppert-Straße 9, 23562 Lübeck, Germany

**Keywords:** Microscopy, Applied optics, Imaging and sensing

## Abstract

Phase-sensitive coherent imaging exploits changes in the phases of backscattered light to observe tiny alterations of scattering structures or variations of the refractive index. But moving scatterers or a fluctuating refractive index decorrelate the phases and speckle patterns in the images. It is generally believed that once the speckle pattern has changed, the phases are scrambled and any meaningful phase difference to the original pattern is removed. As a consequence, diffusion and tissue motion that cannot be resolved, prevent phase-sensitive imaging of biological specimens. Here, we show that a phase comparison between decorrelated speckle patterns is still possible by utilizing a series of images acquired during decorrelation. The resulting evaluation scheme is mathematically equivalent to methods for astronomic imaging through the turbulent sky by speckle interferometry. We thus adopt the idea of speckle interferometry to phase-sensitive imaging in biological tissues and demonstrate its efficacy for simulated data and imaging of photoreceptor activity with phase-sensitive optical coherence tomography. We believe the described methods can be applied to many imaging modalities that use phase values for interferometry.

## Introduction

Phase-sensitive, interferometric imaging measures small changes in the time-of-flight of a light wave by detecting changes in its phase. But in many applications, statistically varying optical properties of the scattering structure randomize the phase of the backscattered light, resulting in a speckle pattern with random intensity and phase^1^. As a consequence, we can only extract meaningful phase differences from images with identical or at least almost identical speckle patterns^2^. However, if the detected wave’s speckle pattern changes over time, it inevitably impedes phase sensitive imaging^3,4^. For example, holographic interferometry or electronic speckle pattern interferometry (ESPI) compare at least two states of backscattered light acquired at different times, and it can only be applied if the respective speckle patterns are still correlated^2–5^. While some techniques, such as dynamic light scattering^6,7^ or optical coherence angiography^8^ make use of these decorrelations, other imaging techniques are severely hindered by it.

Among the most important effects that decorrelate speckles with time, are random motions and changes of the optical path length on a scale below the resolution, e.g., by diffusion^6,7^. These effects are impossible to prevent. But also bulk sample motion can cause the phase evaluation to compare different parts of the same speckle pattern. While in 3D imaging this effect of bulk motion can often be corrected to some extent by co-registration and suitable algorithms, in 2D sectional imaging we lack the data to correct this as the acquired slices of the specimen change if the specimen moves perpendicularly to the imaging plane.

We recently demonstrated imaging of the activity of photoreceptors and neurons by phase-sensitive full-field swept-source optical coherence tomography (FF-SS-OCT)^9,10^. The optical path length of neurons and photoreceptors in the human retina changes by few nanometers upon activation, e.g., after white light stimulation, which can be used as functional contrast for those cellular structures^11–13^. While most OCT systems lack the axial resolution required to resolve this, phases of the OCT signal encode small changes of the optical path length. Using the phases, FF-SS-OCT successfully measured nanometer changes in the optical path length of neurons and receptors due to activation over a few seconds. However, we could neither increase the measurement time nor determine tissue changes in single cross-sectional scans (B-scans). In both cases speckle patterns changed after a few seconds due to diffusion, bulk tissue motion, or tissue deformations and the phase information was lost, ultimately limiting the applicability of the method. Apart from this application, phase evaluation beyond the speckle decorrelation time can be of importance in other fields, such as optical coherence elastography (OCE)^14^, for which Chin *et al*. noticed the importance of phase correlation^15^. Finally, countless applications of OCT make use of the phase^16–19^ and might benefit from better phase evaluation schemes.

**Figure 1:**
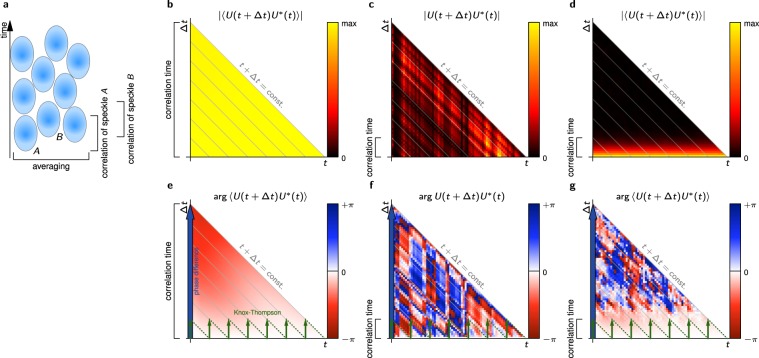
(**a**) In average, each speckle (schematically represented by the blue ellipses) carries valid phase information only for the correlation time of the speckle pattern. Assuming that all speckle in the averaged area are subject to a common phase change in addition to random uncorrelated phase changes, one can use the phase of speckle *A* as long as it is valid and then continue with the phase of speckle *B*. Using multiple speckle, phase information for times significantly exceeding the correlation time can be extracted. (**b**,**e**) Exemplary cross-spectrum magnitude and phase assuming infinite correlation time. Total phase changes can either be computed directly (blue arrow) or using the Knox-Thompson path (green arrows). (**c**,**f**) Magnitude and phase of the cross-spectrum without averaging; neither method can extract the phase beyond the correlation time. (**d**,**g**) Magnitude and phase of ensemble averaged cross-spectrum. Direct phase differences (blue) cannot be used for phase extraction, but Knox-Thompson (green) method can be applied since phase values for small Δ*t* are valid.

In this paper, we reconstruct phase changes over times significantly longer than the decorrelation time of the corresponding speckle patterns. The idea is to calculate phase differences in a series of consecutive images over small time differences (short-time phase differences) that still show sufficient correlation. If this is done for several measurements with different speckle patterns, averaging of these independent measurements will cancel the contributions of the disturbing phase. Integrating all these phase differences then yields the real time evolution of the phase. By averaging the phase in multiple speckles to obtain a single phase value, the real phase change can be obtained beyond the correlation time of the speckles. In essence, we combine the information from multiple speckles, each of which carries information on phase changes over a certain time (see Fig. 1a).

Just integrating the phase difference of successive frames followed by a summation, as it is commonly used for phase unwrapping^16–20^, accumulates the phase noise; except for phase unwrapping it is mathematically equivalent to the direct computation of phase differences. Without averaging, calculating short-time phase differences will not yield any information about phase differences of uncorrelated speckle patterns. The averaging of short-time phase differences before integration is the essential step as it effectively cancels the phase noise.

In the 1970s and 1980s the same idea, known as speckle interferometry, was originally used in astronomy for successful imaging through the turbulent atmosphere with diffraction limited resolution^1,21–28^. Short time exposures were used to reconstruct the missing phase of the Fourier transform of diffraction limited undisturbed images: By utilizing a large number of short-exposure images with different disturbances, true and undisturbed phase differences for small distances in the aperture plane could be computed. From these phase differences, the complete phase was reconstructed by integration. We will frame the similarities and the mathematical analogy between the two methods, which allows us to benefit from advances in astronomy for interferometric measurements during coherent imaging.

Methods that allow phase evaluation beyond the correlation time have also been developed for synthetic aperture radar (SAR) interferometry (InSAR)^29,30^. In InSAR, phase evaluation is used to monitor ground elevation, but small changes on the ground or turbulence in the sky interfere with an evaluation of the phase^29^ similar to interferometric measurements in biological imaging. For InSAR, two techniques have been developed which selectively evaluate the phase from only minimally affected images or structures. The first technique is referred to as small baseline subset technique (SBAS)^31^. It selects optimal images from a time series to compare phase values based on good correlation. The other method only compares phases of single selected scatterers that maintain good correlation over long times, so called permanent scatterers^32^.

In the first part of this paper, we lay down the basics and establish the commonality between coherent phase-sensitive imaging and astronomic speckle interferometry. Afterwards, we demonstrate the efficacy of the resulting methods by simulating simple phase resolved images in backscattering geometry and evaluating these with the proposed approach. Finally, we apply our method to *in* vivo data of phase-sensitive optical coherence tomography.

## Theory

### Mathematical formulation of the problem

We assume coherent imaging of the physical system, in which a deterministic change of the optical path length, e.g., swelling or shrinking of cells, is superimposed by random changes. These may be caused by diffusion, fluctuations of the refractive index, or rapid uncorrelated micro motion. The complex amplitude in each pixel of a coherently acquired and focused image can be represented as a sum of (random) phasors (complex values), where each summand comprises amplitude and phase. Such a coherently acquired field *U*(***x***,*t*) has contributions from the random phases *ϕ*_*i*_(***x***,*t*) and a systematic phase *ϕ*(***x***,*t*). *U*(***x***,*t*) may then be written as

1 $$U({\boldsymbol{x}},t)=\sum _{{\rm{i}}}\,{A}_{i}({\boldsymbol{x}},t){{\rm{e}}}^{{\rm{i}}{\varphi }_{i}({\boldsymbol{x}},t)}{{\rm{e}}}^{{\rm{i}}\varphi ({\boldsymbol{x}},t)},$$

where *A*_*i*_ represents the unknown amplitude of each scatterer, and ***x*** and *t* denote location and time, respectively. Depending on the imaging scenario, *U* might not depend on the location ***x*** at all or ***x*** might be up to three-dimensional as in tomographic imaging.

We can now separate *U* into its systematic phase contribution *U*_0_ and a random modifier *H*, which yields

2 $$\begin{array}{rcl}U({\boldsymbol{x}},t) & = & \mathop{\underbrace{(\sum _{{\rm{i}}}\,{A}_{i}({\boldsymbol{x}},t){{\rm{e}}}^{{\rm{i}}{\varphi }_{i}({\boldsymbol{x}},t)})}}\limits_{H({\bf{x}},t)}\mathop{\underbrace{\,{{\rm{e}}}^{{\rm{i}}\varphi ({\boldsymbol{x}},t)}}}\limits_{{U}_{0}({\boldsymbol{x}},t)}\\  & = & H({\boldsymbol{x}},t){U}_{0}({\boldsymbol{x}},t).\end{array}$$

The modifier *H* covers all effects that alter the speckle patterns over time, e.g., changing phase, changing amplitude, and scatterers moving out of or into the detection area. In this form, we have no way to distinguish *H* from *U*_0_. We can, however, compute the ensemble average by

3 $$\langle U({\boldsymbol{x}},t)\rangle =\langle H({\boldsymbol{x}},t)\rangle {U}_{0}({\boldsymbol{x}},t),$$

when assuming that *U*_0_ (though not *H*) is constant over the averaged ensemble. The ensemble could consist of repeated measurements or certain pixels from a volume, in which *U*_0_ is constant. In this averaged expression, we can now obtain the time evolution of *U*_0_ for times where 〈*H*〉 remains approximately constant, i.e., where the contribution of the random phases is small. For *t* exceeding the correlation time of 〈*H*〉, however, the decorrelation of 〈*H*〉 still prevents us from computing changes in the systematic phase *U*_0_.

### Solving the phase problem in astronomic speckle interferometry

The resolution of large ground-based telescopes is limited by atmospheric turbulences, which modify the phase of the optical transfer function (OTF) and cause time varying speckles in the image. During a long exposure these speckle patterns average out to a blurred image. Speckle interferometry is a way to obtain one diffraction limited image from several short exposure images. In each of these images the random atmospheric turbulences are constant and each image essentially contains one speckle pattern^1^. If the actual diffraction limited image of the object is *I*_0_(***x***) and has a Fourier spectrum ${\mathop{I}\limits^{ \sim }}_{0}({\boldsymbol{k}})$, the Fourier spectra of the short exposure images are given by

4 $$\tilde{I}({\boldsymbol{k}})={ {\mathcal H} }_{S}({\boldsymbol{k}}){\tilde{I}}_{0}({\boldsymbol{k}}),$$

where ${ {\mathcal H} }_{S}$ is the short exposure OTF^1^. ${ {\mathcal H} }_{S}$ represents the disturbing effect of the atmosphere, changes randomly in each acquired image, and causes speckle noise in each image *I*(***x***). A single short exposure image *I*(***x***) will thus not yield the object information because phase and magnitude of ${ {\mathcal H} }_{S}$ are not known. However, the magnitude of the spectrum ${\tilde{I}}_{0}({\boldsymbol{k}})$ of the diffraction limited images is obtained by averaging the squared magnitude of all short exposure spectra $\tilde{I}({\boldsymbol{k}})$,

5 $${\langle |\tilde{I}({\boldsymbol{k}})|\rangle }^{2}=\langle {|{ {\mathcal H} }_{S}({\boldsymbol{k}})|}^{2}\rangle {|{\tilde{I}}_{0}({\boldsymbol{k}})|}^{2},$$

since the squared short exposure OTF averaged over all measurements $\langle {|{ {\mathcal H} }_{S}({\boldsymbol{k}})|}^{2}\rangle$ is larger than zero and can be computed on the basis of known statistical properties of ${ {\mathcal H} }_{S}$^1^. But the magnitude $|{\tilde{I}}_{0}|$of the Fourier spectrum only allows computation of the autocorrelation of *I*_0_. To obtain the full diffraction limited image the phase of ${\tilde{I}}_{0}({\boldsymbol{k}})$ is required too. This phase is contained in the Fourier transforms $\tilde{I}({\boldsymbol{k}})$ of the short time exposures, but it is corrupted by a random phase contribution of the atmospheric turbulences in the same way as the systematic phase in coherent imaging is corrupted by random phase noise.

Hence, retrieving diffraction limited images by speckle interferometry faces the same problem as retrieving the temporal phase evolution in coherent imaging. Only over small spatial frequency distances in the Fourier plane the phase difference in a spectrum $\tilde{I}({\boldsymbol{k}})$ corresponds to the phase difference in the spectrum ${\tilde{I}}_{0}({\boldsymbol{k}})$ of the diffraction limited image. Over larger distances, the phase information is lost due to atmospheric turbulence. The similarity of the problem is seen in the analogy of Eqs (4) with (2), where ${{\mathscr{H}}}_{S}$ and *I* correspond to *H* and *U*, respectively. However, while the astronomic problem has a two-dimensional vector ***k*** of spatial frequencies as the dependent variable, the phase-sensitive imaging problem has only the time *t*. Both use a series of measurements, in which only phase information over small Δ***k*** or Δ*t* is contained, respectively. We will show that by analyzing a large number of measurements, in which the corrupting phase contribution changes, the full phase can be reconstructed.

### Phase information in the cross-spectrum

In astronomy, it was Knox and Thompson who solved the recovery of phases from multiple, statistically varying images^21^ with short exposure times using the so-called cross-spectrum. Later, another approach, called the triple-correlation or bispectrum technique, even improved the achieved results^22^. One major advantage of the bispectrum over the cross-spectrum technique for astronomic imaging is that it is only sensitive to the non-linear phase contributions  of the transfer function ${ {\mathcal H} }_{S}$, since shifted images yield to the same bispectrum and do not pollute the obtained phases (see, for example^23^, for details). However, for phase sensitive imaging, the linear part of the phase evolution of *U*_0_ is generally of interest and thus the bispectrum technique is not applicable here.

Following we will apply the algorithm of Knox and Thompson to recover the phase beyond the speckle correlation time in coherent imaging. For convenience, we will retain the term cross-spectrum, even though in our scenario it is evaluated in time-domain as a function of *t* rather than in (spatial) frequency-domain as a function of ***k***.

We define the cross-spectrum *C*_*H*_ of the phase disturbing speckle modulation *H* by

$${C}_{H}({\boldsymbol{x}},t,{\rm{\Delta }}t)=H({\boldsymbol{x}},t){H}^{\ast }({\boldsymbol{x}},t+{\rm{\Delta }}t).$$

It is subject to speckle noise since *H* itself merely contains speckle (Fig. 1c,f); but its ensemble average

6 $$\langle {C}_{H}({\boldsymbol{x}},t,{\rm{\Delta }}t)\rangle =\langle H({\boldsymbol{x}},t){H}^{\ast }({\boldsymbol{x}},t+{\rm{\Delta }}t)\rangle $$

has in general a magnitude larger than 0 and speckles of *H* are averaged out, at least for small Δ*t*. Most importantly, it is to a good approximation real-valued, i.e., its phase is zero, as long as Δ*t* is within the correlation time of *H*. This real-valuedness was previously shown for the corresponding cross-spectrum $\langle {C}_{{ {\mathcal H} }_{S}}\rangle$ in astronomic imaging^1,27^. For phase imaging, we show the real-valuedness of *C*_*H*_ for small Δ*t* for which the autocorrelation is still strong in the Methods section. If Δ*t* is larger than the correlation time, *H*(***x***,*t*) and H^*^(***x***,*t* + Δ*t*) become statistically independent, the phase of 〈*C*_*H*_〉 gets scrambled and *C*_*H*_ follows speckle statistics.

Now, if we again assume that *U*_0_ and thus ${C}_{{U}_{0}}$ are constant over the ensemble, we compute the averaged cross-spectrum of the measurements of *U* to

7 $$\begin{array}{rcl}\langle {C}_{U}({\boldsymbol{x}},t,{\rm{\Delta }}t)\rangle  & = & \langle U({\boldsymbol{x}},t){U}^{\ast }({\boldsymbol{x}},t+{\rm{\Delta }}t)\rangle \\  & = & \langle {C}_{H}({\boldsymbol{x}},t,{\rm{\Delta }}t)\rangle {C}_{{U}_{0}}({\boldsymbol{x}},t,{\rm{\Delta }}t).\end{array}$$

Knowing that 〈*C*_*H*_〉 is real-valued for small Δ*t*, the phases of 〈*C*_*U*_〉 are determined only by the phases of ${C}_{{U}_{0}}$. If we find phases of *U*_0_ that yield the phases of 〈*C*_*U*_〉 for these small Δ*t*, we obtain the systematic phase *ϕ*(***x***,*t*) we are looking for as introduced in (1).

Note that 〈*C*_*U*_(***x***,*t*,Δ*t*)〉 is related to the time-autocorrelation of *U*, except averaging being performed over the ensemble instead of the time *t* and thus being a function not only of Δ*t* bus also of *t*. Due to this relation, the cross-spectrum maintains comparably large magnitudes for time differences Δ*t* with large autocorrelation values. The magnitudes and phases of exemplary cross-spectra, in the decorrelation-free scenario and with strong decorrelation, with and without the ensemble averaging (obtained from simulations, see Results and Methods) is shown in Fig. 1b–g. The figures illustrate that 〈*C*_*U*_〉 yields a deterministic increase of the phases for small Δ*t* (Fig. 1g) as long as its magnitude (Fig. 1d, related to the autocorrelation) remains large, but only if the ensemble averaging is performed. It can further be seen, that the valid phase differences visible in Fig. 1g are small compared to the phases for large *t* in Fig. 1e since they represent merely phase difference for small Δ*t*. Nevertheless, when reconstructing the phase of *U*_0_ from the values of 〈*C*_*U*_〉, we need to ensure that only values for (small) Δ*t* with strong autocorrelation are taken into account. This condition coincides with the real-valuedness of 〈*C*_*H*_〉.

Ensemble averaging needs multiple independent measurements, which cannot be performed easily in a real experiment. However, averaging over multiple lateral pixels or averaging over multiple detection apertures can be done from a single experiment and approximates the ensemble average. In our case, we either use an area over which we strive to obtain one mean phase-curve, or we use a Gaussian filter with a width determined as a compromise between spatial resolution and sufficient averaging statistics to obtain images of the phase evolution (see Methods).

#### Phase retrieval from the cross-spectrum

Having obtained the cross-spectrum 〈*C*_*U*_〉, the systematic phase function *U*_0_ needs to be extracted. We assume a given initial phase *ϕ*(***x***,*t* = *t*_0_) and evaluate methods to extract the systematic phase evolution *ϕ*(***x***,*t*) from the cross-spectrum: An approach equivalent to computing phase differences directly is given by

8 $$\varphi ({\boldsymbol{x}},{t}_{0}+t)=\varphi ({\boldsymbol{x}},{t}_{0})+{\rm{\arg }}\langle {C}_{U}({\boldsymbol{x}},{t}_{0},t)\rangle ,$$

for *t* ≥ *t*_0_ (blue arrows in Fig. 1b–g). However, it only yields phases within the correlation time. In astronomy, this approach never works, unless the image was not disturbed by turbulence in the first place. Instead, Knox and Thompson originally demonstrated diffraction limited imaging through the turbulent sky^21^ by iteratively walking in small increments of Δ*t* (e.g., one time step) through the cross-spectrum, i.e.,

9 $$\varphi ({\boldsymbol{x}},{t}_{0}+n{\rm{\Delta }}t)=\varphi ({\boldsymbol{x}},{t}_{0}+(n-1){\rm{\Delta }}t)+{\rm{\arg }}\langle {C}_{U}({\boldsymbol{x}},{t}_{0}+(n-1){\rm{\Delta }}t,{\rm{\Delta }}t)\rangle ,$$

for integer *n* ≥ 1 with increasing iteration number *n* (green arrows in Fig. 1b–g). This method uses only phase values well within the correlation time. If 〈*C*_*U*_〉 is valid for the single time steps Δ*t*, i.e., 〈*C*_*H*_〉 is real valued, the iterative formula will yield valid results, even for larger *t*. However, a single outlier at a time *t*_err_, i.e., one false step, ruins all following values for *t* ≥ *t*_err_.

The influence of these single events can be reduced by taking multiple ways with different Δ*t* for stepping through the cross-spectrum. In astronomy, this is known as the extended Knox-Thompson method^23,28^. However, most of the algorithms for finding the optimal paths have been developed for use with the bispectrum technique^24–26^; nevertheless, in general, they can be easily transferred to the cross-spectrum. Here we minimized the sum of weighted squared differences between the measured cross-spectrum and the cross-spectrum resulting from the systematic phase *ϕ*(***x***,*t*). The algorithm is described step-by-step in the methods section.

### Realization of the algorithm

**Figure 2:**
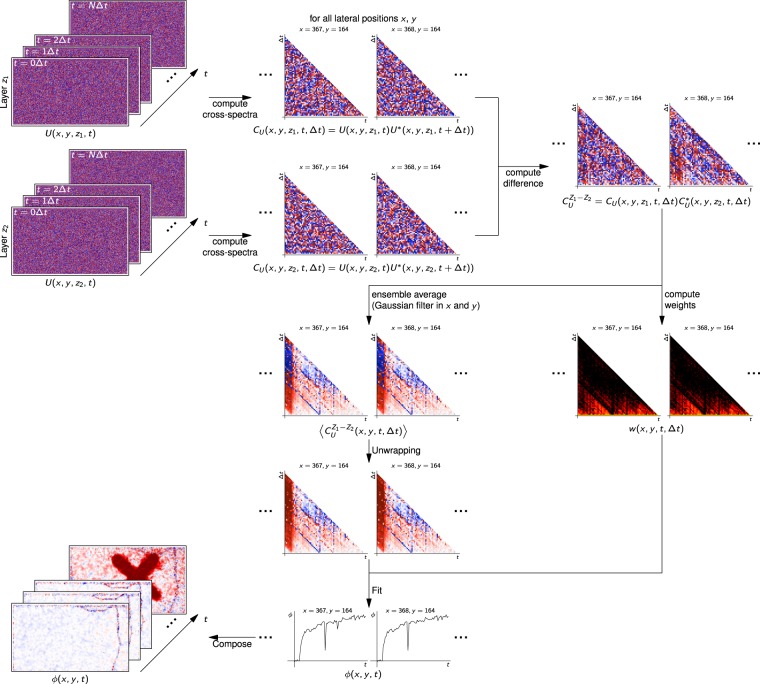
Flowdiagram of the actual implementation of the extended Knox-Thompson algorithm.

To actually implement the presented extended Knox-Thompson algorithm for phase-sensitive OCT imaging we proceeded as follows and as illustrated in Fig. 2. A more detailed description of the required steps is given in the Methods section. We assume the OCT data is reconstructed, co-registered and the layers of interest are segmented and denoted *z*_1_ and *z*_2_, with the volumes being given by *U*(***x***,*t*). For example, these layers can be the inner segment/outer segment junction of the retina and the outer segment tips, respectively, in which case phase difference will show elongation of the photoreceptor outer segments. We now compute the cross-spectra for all points in the respective depths of *z*_1_ and *z*_2_ by

$${C}_{U}({\boldsymbol{x}},t,{\rm{\Delta }}t)=U({\boldsymbol{x}},t){U}^{\ast }({\boldsymbol{x}},t+{\rm{\Delta }}t).$$

To average signals over multiple depths, the complex cross spectra *C*_*U*_ can also be averaged over multiple *z* values here. Next the phase difference between these cross-spectra in the respective layers of interest is performed by computing

$${C}_{U}^{{z}_{1}-{z}_{2}}(x,y,t,{\rm{\Delta }}t)={C}_{U}(x,y,{z}_{1},t,{\rm{\Delta }}t){C}_{U}^{\ast }(x,y,{z}_{2},t,{\rm{\Delta }}t).$$

Utilizing the difference of two layers makes the measurement almost independent from the phase stability of the system (see also Fig. S4 in the Supplementary Information). This step is followed by the actual ensemble average yielding 〈*C*_*U*_〉 as in Eq. (7). To create images, this can be done by applying a Gaussian filter in *x* and *y* direction. In parallel, weights *w*(*x*,*y*,*t*,Δ*t*) are computed that describe the quality of each entry in all cross-spectra and are required to perform a fit afterwards (see Methods section, Eqs (12) and (13)). After phase unwrapping the ensemble averaged cross-spectra 〈*C*_*U*_〉, a phase function is fitted to the phase of 〈*C*_*U*_〉 for each *x* and *y* coordinate, respecting the weights *w*. This yields the phase *ϕ*(*x*,*y*,*t*). These values then compose the resulting images. In case only a single curve is to be extracted, the dependency of *x* and *y* is dropped in the ensemble averaging step and only one curve is fitted.

### Acquisition and reconstruction parameters

The algorithm depends on several acquisition and reconstruction parameters. Among the obvious ones are the number of values used for (ensemble) averaging and the trade-off between obtainable signal-to-noise ratio, e.g., by increasing the integration time of the data acquisition, and the time distance between time-adjacent images. While it is difficult to give definite answers on how to choose these parameters, rough guide-lines can be provided: Accuracy of obtained phases will improve if the averaging size is increased, provided that all averaged data points exhibit the same or at least similar systematic phase changes. In many cases, the practical consideration here will be related to the resolution loss that is accompanied by more averaging or larger filter sizes.

The choice of integration time is ultimately limited by the requirement, that at least temporally adjacent images need to be well correlated. If this is not the case, however, an increased integration time also no longer yields increased signal-to-noise ratio, but rather phase wash-out diminishes the signals. In our cases, we were always limited by technical implications preventing us from decreasing the time between successive measurements and thereby limiting the temporal sampling frequency. However, we are fairly certain that our phase signals would still benefit from more frequent measurements. We believe this holds for most applications since some inherent averaging is done when fitting data to the cross-spectrum in the extended Knox-Thompson method. Exceptions might occur when strong phase noise dominates the cross-spectra.

### Implementation

We implemented the respective algorithms in C++ using the Clang compiler. Vectorization and OpenMP were used to increase performance where possible. Most importantly, to achieve good performance, all cross-spectra in single-precision complex floating point numbers had to fit in memory at the same time, requiring in our case about 10 GB of RAM. Parts requiring linear algebra were implemented using the Armadillo library^33,34^.

## Results

### Simulation

**Figure 3:**
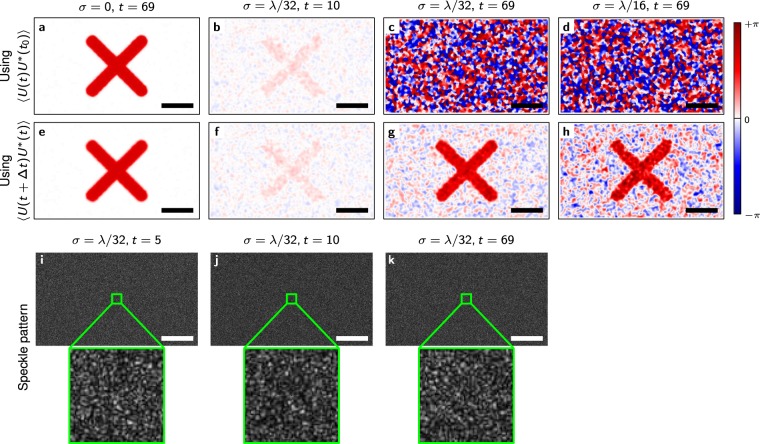
Phase images and speckle patterns obtained from the simulation. (**a–d**) Phase evaluated by phase differences for different *σ* and time steps. (**e–h**) Phase evaluated using the extended Knox-Thompson methods for the same *σ* and time steps as shown in (**a–d**). (**i–k**) Speckle patterns for *σ* = *λ*/32 after a different number of time steps. Scale bars are 500*λ*.

We simulated images of a large number of point scatterers that exhibit a random Gaussian-distributed 3-dimensional motion (with variance *σ*^2^) between frames in addition to a common axial motion in a specific “x”-shaped area. A simulation has the advantage that the actual phase change (ground-truth) is well known and that the speckle decorrelation can be tightly controlled. While both methods show indistinguishable results for the noise-free scenario (*σ* = 0), the simulation in the case of degrading speckle demonstrates the power of the extended Knox-Thompson method. For *σ* = *λ*/32, the phase evaluation with a simple phase difference to the first image and the extended Knox-Thompson method yield almost indistinguishable results for the first 10 steps (Fig. 3b,f). After 60 time steps, a simple phase difference to the first image yields only noise (Fig. 3c), while the “x”-shaped pattern is still well visible (Fig. 3g) using the extended Knox-Thompson method. The speckle patterns appear correlated after 10 steps but are completely changed after 69 steps (Fig. 3i–k). Doubling the scatterer motion to *σ* = *λ*/16, the “x”-shaped pattern begins to deteriorate after 69 steps even with the Knox-Thompson method. However, the “x” remains visible (Fig. 3h), whereas the phase difference again yields only phase noise (Fig. 3d).

**Figure 4:**
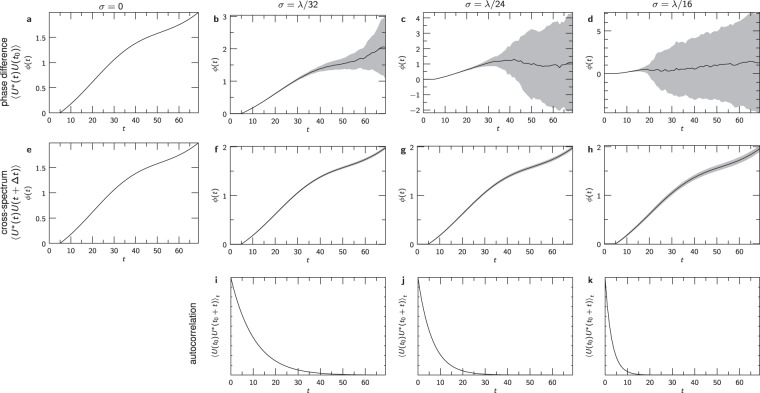
Extracting a single phase curve from simulated images of point scatterers that move between successive measurements by subtracting the phase of the first image (**a**–**d**) and extended Knox-Thompson evaluation of phases (**e**–**h**) while random 3D motion of the simulated scatterers is increased from 0 to *λ*/16. (**i**–**k**) Autocorrelation of temporal changes of the wave field. Each curve was simulated 100×; the grey areas indicate the standard deviation of the obtained values.

The curves extracted in multiple simulations from the “x” confirm the results of the images and demonstrate the reproducibility of the method. Without random scatterer motion (*σ* = 0, Figs 3a,e and 4a,e), the phase can be calculated by the phase difference to the first frame yielding the exact curve that was supplied to the simulation. But introducing random Gaussian-distributed motion of the scatterers with as little as *σ* = *λ*/32 between frames accumulates huge errors in the phase of the directed motion after 70 frames (Fig. 4b); the mean autocorrelation of the complex fields (Fig. 4i) in this case drops to one half in less than ten frames. Increasing motion amplitude (Fig. 4c,d) has devastating effects on the calculated phase differences. Retrieving the phase with the cross-spectrum based Knox-Thompson method yields the directed motion up to *σ* = *λ*/16 (Fig. 4f–h) even though the autocorrelation halves after a few frames (Fig. 4k).

Finally, the dependency on the number of independent samples that are averaged is shown in the Supplementary Information. Fig. S1 shows that smaller averaging numbers increase the fluctuations of the results but do not lead to a general bias. However, for small averaging numbers, the fluctuations increase dramatically. In this simulation, the statistical fluctuations of these averaged and laterally adjacent points should be fully independent. Consequently, these points only share the systematic phase change that we aim to extract.

The resulting curves also show that deviations from the ground truth add up over time, i.e., they increase with time *t*. Although one might expect that, in particular, the extended Knox-Thompson method would be subject to this, it is also clear from Fig. 4 that the effect with the extended Knox-Thompson method is lower than when computing phase differences. The strength of this overall error depends on multiple factors, such as statistical and independent phase noise, how many values are averaged, and on the strength of the (simulated) Brownian motion. Two kinds of effects might contribute to these phase deviations with increasing time *t*. Uncorrelated phase noise that is statistically independent in all acquired or simulated images and decorrelation effects that alter the phases and the speckle with time, but where a correlation remains in successive images. The strong discrepancies observed in Fig. 4 result from decorrelating effects instead of uncorrelated phase noise. The extended Knox-Thompson method cannot improve results if uncorrelated phase noise dominates the images or the data and should in many cases be more vulnerable to this phase noise than the standard phase differences.

To further quantify these results we looked at the average sum of all squared differences of the theoretical curves and the actual curves for all simulated datasets, as well as the difference of the final time-point to the ground-truth curve, which might show accumulated errors (Table 1). As soon as some degree of speckle decorrelation is introduced, the extended Knox-Thompson method outperformed the simple phase differences. For the extended Knox-Thompson method, deviations from the ground truth are orders of magnitude lower than for simple phase differences.

**Table 1:** Quantitative comparison of the simulated results (compare Fig. 4).

	Mean over all *t*		Final *t*	
	Phase differences	Extended Knox-Thompson	Phase differences	Extended Knox-Thompson
*σ* = 0	0.0 ± 0.0	0.0 ± 0.0	0.0 ± 0.0	0.0 ± 0.0
*σ* = *λ*/32	0.17 ± 0.38	0.00038 ± 0.00042	2.8 ± 6.6	0.0010 ± 0.0012
*σ* = *λ*/24	2.1 ± 1.3	0.00068 ± 0.00082	7.3 ± 8.1	0.0015 ± 0.0020
*σ* = *λ*/16	3.7 ± 1.3	0.0024 ± 0.0031	6.8 ± 7.8	0.0054 ± 0.0073

Values indicate the mean squared errors over the simulated datasets taken all values (mean over all *t*) or only the final values in a time series (final *t*) into account.

### *In vivo* experiments

**Figure 5:**
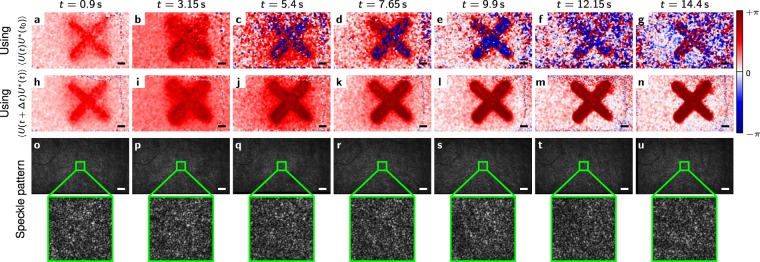
Phase differences between the ends of the photoreceptor outer segment in the living human eye at different times compared to the initial phase at *t* = 0. The entire measurement ended at about *t* = 14.6 s after initiating a light stimulus. (**a–g**) Phase difference obtained by directly comparing to pre-stimulus phase with phase after different times. (**h–n**) Phase difference obtained from the cross-spectrum as described in the Methods section (by minimizing Eq. (14)). (**o–u**) Speckle pattern of one of the involved layers before the stimulus and at the respective times. It can clearly be seen, that that the speckle pattern changes with time. Scale bars are 200 *μ*m.

The *in vivo* experiments confirm the simulation results. Here, we imaged human retina with full-field swept-source optical coherence tomography (FF-SS-OCT, see Methods), which provides three-dimensional tomographic data comprising amplitude and phase, to show the elongation of photoreceptor cells in the living human retina. Phase differences between both ends of the photoreceptor outer segments were evaluated before and during stimulating the photoreceptors with an “x”-shaped light-stimulus, similar to the simulation (see Methods). Figure 5a–g show the extracted phase difference of the 5th frame (the beginning of the stimulus) to various other frames of the data set. The last frame was acquired 14.6 seconds later. After 5 seconds the phase differences are dominated by random phases and the speckle patterns of the compared images changed considerably after this time (Fig. 5o–u). Diffusion and uncorrected tissue motion are likely to be the dominant factors. The phase evaluation using the cross-spectrum is nevertheless able to reconstruct the phase difference and show the elongation of the photoreceptor outer segments (Fig. 5h–n). Even in the non-stimulated area of Fig. 5g, it is clearly seen that the phase difference gives random results, whereas the extended Knox-Thompson approach shows the expected small changes (Fig. 5n). It is also worth mentioning that the phase differences wrap causing the blue colour within the stimulated area in Fig. 5c–g. In contrast to this, the values of the extended Knox-Thompson do not wrap but are clipped at +*π* in the false-colour representation of Fig. 5j–n.

A second measurement with a brief stimulation lasting 200 ms that was taken over 8 seconds is shown in Fig. S3. This measurement demonstrates the relaxation of the signals. It is visible that both methods, using phase differences and the extended Knox-Thompson method, yield comparable results here, albeit the extended Knox-Thompson method shows reduced noise in the resulting phase images. Small residual signals remain in either method.

Finally, we also compared phase results for different averaging parameters (Supplementary Document Fig. S2), represented by the size of the Gaussian filter in Eq. (11). The results show that even for small averaging of laterally adjacent pixels the extended Knox-Thompson method outperforms simple phase differences. Contrary to the simulated case, lateral pixels might still show some correlation in their phase fluctuations since the actual motion is unknown and there might be systematic motion that is correlated in regions smaller than the filter size. The results indicate, however, that this does not result in major problems for the extended Knox-Thompson algorithm.

While uncorrelated phase noise is inevitable using FF-SS-OCT, it is not dominating the image degradation in Fig. 5a–g. Rather, major improvements in the *in vivo* experiments are visible when using the extended Knox-Thompson method, which indicates that decorrelating speckle patterns and phases dominate the imaging scenario and these originate from the specimen, i.e., the retina in these images.

### Processing times

Obviously, the extended Knox-Thompson method has additional computational cost compared to the simple phase differences. Table 2 lists the required execution times for a single dataset as shown in Figs 3–5 as measured on an Intel Core i7 8700 with 32 GB of RAM. Although the extended Knox-Thompson method requires a processing time exceeding the phase differences by up to 2 orders of magnitude, processing times remain manageable with under 2 minutes in all tested cases. For our specific imaging scenario, this time is negligible when compared to the required pre-processing time (see Methods), comprising reconstruction, motion correction, registration and segmentation, which takes hours for one dataset.

**Table 2:** Computational time required to evaluate single datasets of the data shown in Figs 3–5, for both simulated and experimental data.

	Simulated data		Experimental data
	Image (Fig. 3)	Curve (Fig. 4)	Image (Fig. 5)
Standard	0.34 ± 0.08s	0.27 ± 0.06s	2.33 ± 0.06s
Extended Knox-Thompson	109 ± 6s	40 ± 1s	109 ± 2s

## Conclusion

Building the phase differences of successive frames, and then summing or integrating the differences has been used in phase sensitive imaging^16–20^. It removes the burden of phase unwrapping and thereby improves results. In contrast, including an average over multiple speckles after computing the phase differences and before integrating those differences again, as done by the Knox-Thompson method in astronomic speckle interferometry, allows phase sensitive imaging over more than ten times the speckle decorrelation time. To our knowledge, this important step has never been fully realized in phase sensitive imaging. The resulting method overcomes limitations from decorrelating speckle patterns and recovers phase differences even if speckle are completely decorrelated. Using extended Knox-Thompson methods yields the optimized results presented here and removes the sensitivity to outliers. The method achieves this by attempting to separate random phase and speckle decorrelations (such as Brownian motion) and systematic phase changes. This requires some form of presumptions on the data, e.g., on the spatial dependence of the systematic changes, that will influence the results of the algorithm and inaccurate assumptions introduce some form of error into the results. But, since simple phase differences do not yield any meaningful results after decorrelation of the speckles, the proposed extended Knox-Thompson method is an improved tool for phase sensitive imaging in these scenarios.

Still, measurements and the algorithm can be further improved in several regards. We approximated the ensemble average either by averaging a certain area of lateral pixels or by a Gaussian filter. The former is suitable to obtain the mean expansion in a certain area, the latter is used to obtain images. But to get high-resolution images, using spatial filtering is not ideal. Other speckle averaging techniques, e.g., based on non-local means^35,36^, and time-encoded manipulation of the speckle pattern by deliberately manipulating the sample irradiation (similar to^37^) should preserve the full spatial resolution.

Increasing the sampling rate of the *t* axis of the cross-spectrum, i.e., decreasing the smallest Δ*t*, should also improve results, since then the correlation of compared speckle patterns is improved. The results presented here are limited by the finite sampling interval of the *t*-axis and not by the overall measurement time; however, at some point, it is expected that errors add up for high frequent sampling of *t* as data size increases further. Alternatively, one could acquire two immediate frames with a small Δ*t*, followed by a larger time gap to the next two frames (during which speckle patterns can begin to decorrelate). The immediate frames can give the correct current rate of phase changes (the first derivative of the phase change) that can then be extrapolated linearly to the time of the next two frames, etc. Obviously, this can be extended to three or more frames with small Δ*t* giving the second or even higher order local derivative, respectively.

There are also extensions of the presented method possible. For example, the extraction of systematic phase changes in a dynamic, random speckle pattern can be combined with dynamic light scattering (DLS)^6,7^ basically separating the random diffusion from systematic particle motion. This approach might give additional contrast compared to either method on its own.

Overall, applications of phase sensitive imaging over long times are manifold. Applications range from biological phase imaging to measure retinal pulse waves^19^, detect cellular activity^9,10^, all the way to visualize deformations using electronic speckle interferometry (ESPI). In particular, for OCT imaging of neuron function of layers that are more severely decorrelating and have lower signal-to-noise ratio compared to the photoreceptors, the proposed method will be of importance.

## Methods

### Simulation

For the simulation we created a complex valued image series with 640 × 368 pixels and a pixel spacing of 4*λ*, where *λ* is the simulated light wavelength. To obtain an image series we created a collection of 50 × 640 × 368 = 11,776,000 point scatterers each getting a random *x*, *y*, and *z* coordinate, as well as an amplitude *A*. While the *x* and *y* coordinate was distributed entirely random in the image area, the *z* coordinate was restricted between 0 and 10*λ* and the amplitude was equally distributed between 0 and 1. To create an image we iterated over all scatterers, and summed the values *A*exp(i2*kz*) for the pixel corresponding to the scatterer’s *x* and *y* coordinate, where *z* is the scatterer’s *z*-value, and *k* = 2*π*/*λ*. This basically corresponds to a phase one would obtain in reflection geometry when neglecting any possible defocus. Finally the image was laterally filtered simulating a limited numerical aperture (NA).

For each simulation we created a series of 70 images; starting with the initial scatterers, to create the next frame, we moved each scatterer randomly as specified by a Gaussian distribution with a certain variance *σ*^2^ in *x*, *y*, and *z* direction. Furthermore, all scatterers currently having *x* and *y*-coordinates as found in a pre-created “x”-shaped mask were additionally subjected to the movement

$${\rm{\Delta }}z=\{\begin{array}{cc}\frac{\lambda }{400}+\frac{\lambda }{800}\,\sin (0.1\,(t-{t}_{0})) & t\ge {t}_{0}\\ 0 & t < {t}_{0}\end{array},$$

where *t* is the frame number and *t*_0_ = 5 in each frame.

### Experiments

**Figure 6:**
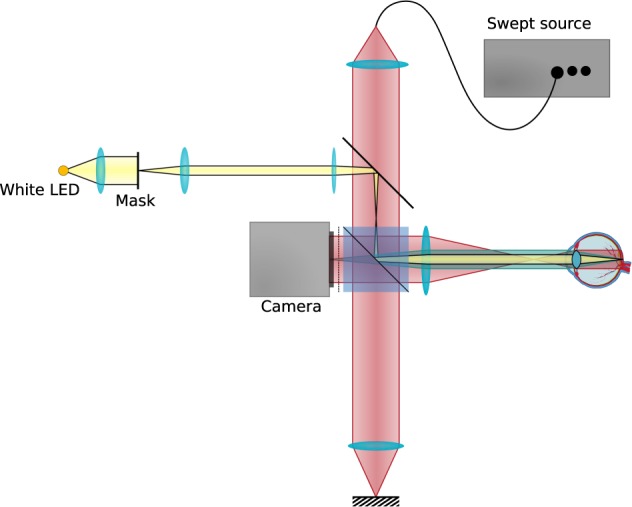
Full-field swept-source optical coherence tomography setup used for acquiring *in vivo* data.

*In vivo* data was acquired with a Michelson interferometer-based full-field swept-source optical coherence tomography system (FF-SS-OCT) as shown in Fig. 6. The setup is similar to the system previously used for full-field and functional imaging^9,19,38^. Light from a Superlum Broadsweeper BS-840-1 is collimated and split into reference and sample arm. The reference arm light is reflected from a mirror and then directed by a beam splitter onto a high-speed area camera (Photron FASTCAM SA-Z). The sample light is directed in such a way, that it illuminates the retina with a collimated beam; the light backscattered by the retina is imaged through the illumination optics and the beamsplitter onto the camera, where it is superimposed with the reference light.

In addition the retina was stimulated with a white LED, where a conjugated plane to the retina contained an “x”-shaped mask. A low-pass optical filter in front of the camera ensured that the stimulation light does not reach the camera.

Swept laser, camera, and stimulation LED were synchronized by an Ardunio Uno microprocessor board. It generates the trigger signals such that the laser sweeps 70× for one dataset. During each sweep the camera acquires 512 images with 640 × 368 pixels each at a framerate of 60,000 frames/s. Each set of 512 images corresponds to one OCT volume. The stimulation LED is triggered after the first 5 volumes and remains active for the rest of the dataset. The time between volumes determines the overall measurement time. The number of 70 volumes was limited by the camera’s internal memory since the acquisition of the camera is too fast to stream the data to a computer in real time.

Measurement light had a centre wavelength of 841 nm and a bandwidth of 51 nm. About 5 mW of this light reached the retina illuminating an area of about 2.6 mm × 1.5 mm, which is the area imaged onto the camera. The “x”-pattern stimulation had significantly less irradiant power, of about 20 *μ*W white light.

#### Phase stability

Additionally, we characterized the phase stability of the setup by measuring a cover slip glass that contains two reflecting surfaces over almost 30 seconds. As shown in the Supplementary Information (Fig. S4), the phase observed in a single lateral position in one of the two surfaces of the cover slip fluctuates with time. As a consequence, the autocorrelation of this single layer on its own decreases significantly after a few seconds. Taking the phase difference of both layers shows excellent phase stability and autocorrelation over the entire measurement time. Our measurements are thus limited by phase decorrelation of the specimen and not by the phase stability of the system.

#### *In vivo* data acquisition

All investigations were done with healthy volunteers; written informed consent was obtained from all subjects. Compliance with the maximum permissible exposure (MPE) of the retina and all relevant safety rules was confirmed by the responsible safety officer. The study was approved by the ethics board of the University of Lübeck (ethics approval Ethik-Kommission Lübeck 16-080).

#### Data reconstruction

Data was reconstructed, registered and segmented as described in previous publications^10^: After a background removal the OCT signal was reconstructed by a fast Fourier transform (FFT) along the spectral axis. Next step was a dispersion correction by multiplication of the complex-valued volumes in the axial Fourier domain (after an FFT along the depth axis) with a correcting phase function that was determined iteratively by an optimization of sharpness metric of the OCT images^38^. Co-registering the volumes aligned the same structures in the same respective voxels of the data series as precisely as the present and changing speckle patterns permitted. This registration also took other layers of the retina into account making use of all retinal structures. Finally, segmenting the average volume allowed aligning of the photoreceptor layers in a certain constant depth for easier phase extraction.

### Phase evaluation

#### Computing the cross-spectrum

To compute the cross-spectrum, we took the series of OCT volumes *U*(***x***,*t*) and computed the cross-spectrum by

$${C}_{U}({\boldsymbol{x}},t,{\rm{\Delta }}t)=U({\boldsymbol{x}},t){U}^{\ast }({\boldsymbol{x}},t+{\rm{\Delta }}t).$$

#### Comparing phases of two different depths

For the *in vivo* experiments we additionally want to compare the phases between different ranges of layers. Let *Z*_1_ and *Z*_2_ denote the sets of layers, then we computed the effective phase difference cross-spectrum by

10 $${C}_{U}^{{Z}_{1}-{Z}_{2}}(x,y;t,{\rm{\Delta }}t)={\textstyle (}\sum _{z\in {Z}_{1}}\,{C}_{U}(x,y,z;t,{\rm{\Delta }}t){\textstyle )}{{\textstyle (}\sum _{z\in {Z}_{2}}{C}_{U}(x,y,z;t,{\rm{\Delta }}t){\textstyle )}}^{\ast },$$

where we now represented the vector ***x*** by its components (*x*,*y*,*z*). For the simulation, there is only one layer to be compared, and thus we set the phase difference cross-spectrum equal to the cross-spectrum of this one layer, i.e., ${C}_{U}^{{Z}_{1}-{Z}_{2}}={C}_{U}$.

#### Approximating the ensemble average

To extract a curve the phase difference was approximated by averaging the cross-spectrum over the *x* and *y* coordinates belonging to a mask *M*, i.e.,

$${\langle {C}_{U}(t,{\rm{\Delta }}t)\rangle }_{M}\approx \frac{1}{|M|}\sum _{(x,y)\in M}\,{C}_{U}^{{Z}_{1}-{Z}_{2}}(x,y;t,{\rm{\Delta }}t),$$

where |*M*| is the number of pixels in the mask *M*.

To obtain images, instead, the ensemble average was approximated by applying a Gaussian filter (convolving with a Gaussian ${G}_{{\sigma }_{{\rm{Gauss}}}^{2}}$ with variance ${\sigma }_{{\rm{Gauss}}}^{2}$):

11 $$\langle {C}_{U}(x,y;t,{\rm{\Delta }}t)\rangle \approx {G}_{{\sigma }_{{\rm{Gauss}}}^{2}}(x,y)\ast {C}_{U}^{{Z}_{1}-{Z}_{2}}(x,y;t,{\rm{\Delta }}t),$$

where * is the convolution in *x* and *y* direction. In effect we used a circular convolution by using a fast Fourier transform (FFT) based algorithm. For Figs 3 and 5 a Gaussian filter of width *σ*_Gauss_ = 4 pixels was used. A comparison of different sizes is found in the Supplementary Information in Fig. S2. For Fig. 4 ensemble averaging was performed over the entire ‘*x*’-shaped pattern, containing 26,437 independent data points. The dependency on the number of data points for curves is shown in the Supplementary Information (Fig. S1).

#### The weights

We additionally used weights to specify how reliable the respective value of the cross-spectrum 〈*C*_*U*_〉 is. Unfortunately, most algorithms that were used previously to compute weights in astronomy cannot be computed as efficiently as 〈*C*_*U*_〉, since we used a fast convolution to apply the Gaussian filter in Eq. (11). We therefore use the weights

12 $$w(t,{\rm{\Delta }}t)=T{\textstyle (}\sum _{(x,y)\in M}\,\frac{{C}_{U}^{{Z}_{1}-{Z}_{2}}(t,{\rm{\Delta }}t)}{|{C}_{U}^{{Z}_{1}-{Z}_{2}}(t,{\rm{\Delta }}t)|}-b{\textstyle )}$$

for phase curve extraction and

13 $$w(x,y;t,{\rm{\Delta }}t)=T{\textstyle (}{\textstyle [}{G}_{{\sigma }_{{\rm{G}}{\rm{a}}{\rm{u}}{\rm{s}}{\rm{s}}}^{2}}(x,y)\ast \frac{{C}_{U}^{{Z}_{1}-{Z}_{2}}(x,y;t,{\rm{\Delta }}t)}{|{C}_{U}^{{Z}_{1}-{Z}_{2}}(x,y;t,{\rm{\Delta }}t)|}{\textstyle ]}-b{\textstyle )}$$

for phase imaging; both could be computed as efficiently as 〈*C*_*U*_〉 itself. In these formula *T* is a cut-off function for negative values:

$$T(x)=\{\begin{array}{cc}x & {\rm{f}}{\rm{o}}{\rm{r}}\,x\ge 0\\ 0 & {\rm{f}}{\rm{o}}{\rm{r}}\,x < 0\end{array}$$

The truncation of negative values and the introduction of *b* are supposed to keep the expectation value for randomly distributed *C*_*U*_ at 0. The sum in (12) and the convolution term in (13) follow speckle statistics if the phase of *C*_*U*_ is random, since they are a sum of random phasors^1^. Consequently, a non-zero value is expected. This can be used to determine suitable values for *b*. The amplitude probability of a speckle pattern is given by a Rayleigh distribution^1^

$$p(A)=\frac{A}{{\sigma }^{2}}\exp {\textstyle (}-\frac{{A}^{2}}{2{\sigma }^{2}}{\textstyle )}.$$

Enforcing a certain threshold *A*_0_ gives a total probability

$${P}_{0}({A}_{0})={\int }_{0}^{{A}_{0}}\,{\rm{d}}A^{\prime} \,p(A^{\prime} )=1-{{\rm{e}}}^{-\frac{{A}_{0}^{2}}{2{\sigma }^{2}}}$$

and its inverse

$${A}_{0}({P}_{0})=\sqrt{-2{\sigma }^{2}\,\mathrm{ln}(1-{P}_{0})}.$$

With this formula, we selected *b* to be the 99% threshold, i.e., *b* = *A*_0_(0.99)), except for Fig. 3 for which *b* = *A*_0_(0.99999) was chosen.

For the two scenarios of curve extraction and imaging with a Gaussian convolution, the parameter *σ* of the Rayleigh distribution remains to be determined. Starting with the derivation of speckle statistics^1^, it can be computed to yield

$$\sigma =\frac{1}{\sqrt{2|M|}}$$

for curve extraction and

$$\sigma =\frac{1}{\sqrt{8\pi }{\sigma }_{{\rm{Gauss}}}}$$

for imaging, when assuming unit magnitude phasors (as present in (12) and (13)) with completely random phases.

#### Phase unwrapping of the cross-spectrum

While alternate approaches to extract the phase from the cross-spectrum deal with the phase wrapping problem differently (e.g.^26^), for our scenario phases of the obtained cross-spectrum need to be unwrapped in the Δ*t* axis in order to obtain good results. However, in 1D phase unwrapping, single outliers, i.e., a random phase for single specific Δ*t*, can tremendously degrade results for all following values. We therefore slightly modified the standard 1D-phase unwrapping approach:

We assume the initial *D* phases to be free of wrapping. Afterwards we moved through the data set from small Δ*t* to larger Δ*t*. We computed the sum of all absolute values of the phase differences to the preceding *D* phases for each phase value that is to be determined. We increased or decreased the respective phase value by 2*π* as long as this difference sum kept decreasing. Then me move to the next Δ*t*. This procedure was done once for curve extraction and repeated for each *x* and *y* value for imaging. In our scenario, we used *D* = 10.

#### Obtaining the phase from the cross spectrum

Instead of using the approaches described by (8) or (9), we formulate the recovery of the phases from the cross-spectrum as a linear least squares problem. This also served as basis for many of the previously demonstrated approaches in astronomy. Assuming the phase of the cross-spectrum is unwrapped in its Δ*t* axis, we can assume that

14 $$\sum _{t,{\rm{\Delta }}t}\,{w}^{2}({\boldsymbol{x}};t,{\rm{\Delta }}t){|\varphi ({\boldsymbol{x}},t+{\rm{\Delta }}t)-\varphi ({\boldsymbol{x}},t)-\langle {C}_{U}({\boldsymbol{x}};t,{\rm{\Delta }}t)\rangle |}^{2}$$

minimizes for the desired phase *ϕ*(***x***,*t*), if the weights *w* represent the quality of the cross-spectrum for the respective parameters ***x***, *t*, and Δ*t*. However, the solution to this linear least square problem is not unique: since the cross-spectrum only contains phase differences, there will be different solutions for different initial values *ϕ*(***x***,*t* = 0). In addition, all weights might be 0 for one specific *t* which represents a gap in reliable data. Both problems in the evaluation can be solved by Tikhonov regularization with small parameters. For the former case one should force the resulting phase for one time point *t*_0_ to be small; the corresponding regularization parameter should not influence other results as long as it is chosen sufficiently small to not introduce numerical errors. For the second problem, a difference regularization can be introduced. Again, the parameter can be small; it is only used is to obtain a unique solutions, in case weights approach 0. Consequently, the entire approach can be formulated as a linear least squares problem, and solved by regularized solving of the respective linear equation.

To solve the least squares problem (14) we first need to discretize it properly. To this end, we first create the discrete vectors and matrices ***c****ϕ* corresponding to the unwrapped cross-spectrum phase arg〈(*C*_*U*_)〉, ***ϕ*** corresponding to the phases to be extracted, and a matrix *S* relating the two. Assume a given systematic phase ***ϕ***′, then the corresponding cross-spectrum ***c***_*ϕ*_′ would be uniquely given by

$${{\bf{c}}{}^{{\boldsymbol{^{\prime} }}}}_{\varphi }=(\begin{array}{c}{{\varphi }^{{\rm{^{\prime} }}}}_{0}-{{\varphi }^{{\rm{^{\prime} }}}}_{1}\\ {{\varphi }^{{\rm{^{\prime} }}}}_{0}-{{\varphi }^{{\rm{^{\prime} }}}}_{2}\\ \vdots \\ {{\varphi }^{{\rm{^{\prime} }}}}_{1}-{{\varphi }^{{\rm{^{\prime} }}}}_{2}\\ \vdots \end{array})=\mathop{\underbrace{(\begin{array}{ccccc}1 & -1 & 0 & \cdots  & 0\\ 1 & 0 & -1 & \cdots  & 0\\  &  & \vdots  & \ddots  & \\ 0 & 1 & -1 & \cdots  & 0\\  &  & \vdots  &  & \end{array})}}\limits_{S}(\begin{array}{c}{{\varphi }^{{\rm{^{\prime} }}}}_{0}\\ {{\varphi }^{{\rm{^{\prime} }}}}_{1}\\ \vdots \\ {{\varphi }^{{\rm{^{\prime} }}}}_{N-1}\end{array}).$$

We can write this as

$${{\bf{c}}{\boldsymbol{^{\prime} }}}_{\varphi }=S{\boldsymbol{\varphi }}\text{'}.$$

Given the actually measured cross-spectrum ***c***_*ϕ*_ and the corresponding phase ***ϕ*** to be computed and introducing the diagonal weight matrix *W* = diag(*w*_0_,*w*_1_, …, *w*_*N*−1_) as computed by (12) or (13) we can write the determination of ***ϕ*** as the regularized minimization problem by

$${\Vert W(S\varphi -{c}_{\varphi })\Vert }^{2}+{\mu }_{1}{\Vert {{\rm{\Gamma }}}_{1}\varphi \Vert }^{2}+{\mu }_{2}{\Vert {{\rm{\Gamma }}}_{2}\varphi \Vert }^{2}+{\mu }_{3}{\Vert {{\rm{\Gamma }}}_{3}\varphi \Vert }^{2}\to \,{\rm{\min }}\,.$$

The corresponding ***ϕ*** is found by

$${\boldsymbol{\varphi }}={({S}^{T}{W}^{2}S+{\mu }_{1}^{2}{{\rm{\Gamma }}}_{1}^{T}{{\rm{\Gamma }}}_{1}+{\mu }_{2}^{2}{{\rm{\Gamma }}}_{2}^{T}{{\rm{\Gamma }}}_{2}+{\mu }_{3}^{2}{{\rm{\Gamma }}}_{3}^{T}{{\rm{\Gamma }}}_{3})}^{-1}\,{S}^{T}{W}^{2}{{\bf{c}}}_{\varphi },$$

which needs to be performed for each curve or each lateral pixel (*x*,*y*) when doing phase imaging. In general, we chose

$${{\rm{\Gamma }}}_{1,ij}=\{\begin{array}{cc}1 & {\rm{f}}{\rm{o}}{\rm{r}}\,i={\rm{j}}=4\\ 0 & {\rm{o}}{\rm{t}}{\rm{h}}{\rm{e}}{\rm{r}}{\rm{w}}{\rm{i}}{\rm{s}}{\rm{e}}\end{array},$$

$${{\rm{\Gamma }}}_{2,ij}=\{\begin{array}{cc}1 & {\rm{i}}{\rm{f}}\,i={\rm{j}}\ne 0\\ -1 & {\rm{i}}{\rm{f}}\,i={\rm{j}}+1\\ 0 & {\rm{o}}{\rm{t}}{\rm{h}}{\rm{e}}{\rm{r}}{\rm{w}}{\rm{i}}{\rm{s}}{\rm{e}}\end{array},$$

and

$${{\rm{\Gamma }}}_{3,i,j}=\{\begin{array}{cc}2 & {\rm{i}}{\rm{f}}\,i={\rm{j}},i\ne 0,\,{\rm{a}}{\rm{n}}{\rm{d}}\,i\ne N-1\\ -1 & {\rm{i}}{\rm{f}}\,i=j+1\,{\rm{o}}{\rm{r}}\,i={\rm{j}}-1\\ 0 & {\rm{o}}{\rm{t}}{\rm{h}}{\rm{e}}{\rm{r}}{\rm{w}}{\rm{i}}{\rm{s}}{\rm{e}}\end{array},$$

with *μ*_1_ = 0.02, *μ*_2_ = 0.0001, and *μ*_3_ = 0.02. Without the regularization terms, the matrix *S* is not invertible or not invertible if there are gaps in the data.

#### Specifying the initial value

The regularization term ${\mu }_{1}{\Vert {{\rm{\Gamma }}}_{1}\Vert }^{2}$ basically enforces the phase value corresponding to the 5th volume to 0, thereby specifying the initial value. Since in both, simulation and experiment, we only know that no (deliberate) phase change is occurring in the frames <5, we can normalize the final result by

$${\varphi }_{{\rm{res}},i}={\varphi }_{i}-\frac{1}{{N}_{0}}\mathop{\sum }\limits_{j=0}^{{N}_{0}-1}\,{\varphi }_{j},$$

with *N*_0_ = 5.

#### Real-valuedness of the modulating cross-spectrum

For the entire approach to work it remains to be shown, that the modulation cross spectrum 〈*C*_*H*_〉 is real-valued for small Δ*t*. We assume the modulation cross-spectrum is given according to (2) and (6) by

$$\begin{array}{rcl}\langle {C}_{H}\rangle  & = & \langle H(t){H}^{\ast }(t+{\rm{\Delta }}t)\rangle \\  & = & \langle (\sum _{i}\,{A}_{i}(t){{\rm{e}}}^{{\rm{i}}{\varphi }_{i}(t)}){(\sum _{j}{A}_{i}(t+{\rm{\Delta }}t){{\rm{e}}}^{{\rm{i}}{\varphi }_{i}(t+{\rm{\Delta }}t)})}^{\ast }\rangle \\  & = & \langle \sum _{i}\,{A}_{i}(t){A}_{i}^{\ast }(t+{\rm{\Delta }}t){{\rm{e}}}^{{\rm{i}}({\varphi }_{i}(t)-{\varphi }_{i}(t+{\rm{\Delta }}t))}+\sum _{i,j,i\ne j}\,{A}_{i}(t){A}_{j}^{\ast }(t+{\rm{\Delta }}t){{\rm{e}}}^{{\rm{i}}({\varphi }_{i}(t)-{\varphi }_{j}(t+{\rm{\Delta }}t))}\rangle \\  & = & \langle \sum _{i}\,{A}_{i}(t){A}_{i}^{\ast }(t+{\rm{\Delta }}t){{\rm{e}}}^{{\rm{i}}({\varphi }_{i}(t)-{\varphi }_{i}(t+{\rm{\Delta }}t))}\rangle +\langle \sum _{i,j,i\ne j}\,{A}_{i}(t){A}_{j}^{\ast }(t+{\rm{\Delta }}t){{\rm{e}}}^{{\rm{i}}({\varphi }_{i}(t)-{\varphi }_{j}(t+{\rm{\Delta }}t))}\rangle .\end{array}$$

Now, the second term is small compared to the first term since *ϕ*_*i*_ and *ϕ*_*j*_ are statistically independent making the sum and the average run over random phasors. The phases of the first term can be approximated by *ϕ*_*i*_(*t*) − *ϕ*_*i*_(*t*) + ∂_*t*′_*ϕ*_*i*_(*t*′)|_*t*′=*t*_ Δ*t* = ∂_*t*′_*ϕ*_*i*_(*t*′)|_*t*′=*t*_ Δ*t* and thus will be 0 for small Δ*t* that are within good autocorrelation of the modulating function *H*. Thus 〈*C*_*H*_〉 is real for small Δ*t* to a good approximation.

### Phase evaluation by phase differences

In the alternative approach we compute merely phase differences. We can still formulate this by using the cross-spectrum of phase difference ${C}_{U}^{{Z}_{1}-{Z}_{2}}$ given by (10). For all values *t* ≥ *t*_0_, the phase is obtained by

$$\varphi (t)=\arg \{{G}_{{\sigma }_{{\rm{G}}{\rm{a}}{\rm{u}}{\rm{s}}{\rm{s}}}^{2}}\ast {C}_{U}^{{Z}_{1}-{Z}_{2}}(x,y;{t}_{0},t)\}$$

for imaging and

$$\varphi (t)={\rm{\arg }}\sum _{(x,y)\in M}\,{C}_{U}^{{Z}_{1}-{Z}_{2}}(x,y;{t}_{0},t)$$

for curve evaluation. For this direct phase computation, the phase-wrapped cross-spectrum was used. For curve extraction the phase was unwrapped in *t* afterwards.

## Supplementary information

Supplementary information

## Data Availability

The datasets generated and/or analysed during the current study are available from the corresponding author on reasonable request.

## References

[1] Goodman, J. *Speckle Phenomena in Optics: Theory and Applications* (Roberts & Company, 2007).

[2] Creath K (1985). Phase-shifting speckle interferometry. Appl. Opt..

[3] Joenathan C, Haible P, Tiziani HJ (1999). Speckle interferometry with temporal phase evaluation: influence of decorrelation, speckle size, and nonlinearity of the camera. Appl. Opt..

[4] Lehmann M (1997). Decorrelation-induced phase errors in phase-shifting speckle interferometry. Appl. Opt..

[5] Jones R, Wykes C (1977). De-correlation effects in speckle-pattern interferometry. Opt. Acta.

[6] Berne, B. & Pecora, R. *Dynamic Light Scattering: With Applications to Chemistry, Biology, and Physics. Dover Books on Physics Series* (Dover Publications, 2000).

[7] Goldburg WI (1999). Dynamic light scattering. Am. J. Phys.

[8] Chen C-L, Wang RK (2017). Optical coherence tomography based angiography [invited]. Biomed. Opt. Express.

[9] Hillmann D (2016). *In vivo* optical imaging of physiological responses to photostimulation in human photoreceptors. Proc. Natl. Acad. Sci. USA.

[10] Pfäffle, C. *et al*. Functional imaging of ganglion and receptor cells in living human retina by osmotic contrast. ArXiv e-prints 1809.02812, https://arxiv.org/abs/1809.02812 (2018).

[11] Zhang Pengfei, Zawadzki Robert J., Goswami Mayank, Nguyen Phuong T., Yarov-Yarovoy Vladimir, Burns Marie E., Pugh Edward N. (2017). In vivo optophysiology reveals that G-protein activation triggers osmotic swelling and increased light scattering of rod photoreceptors. Proceedings of the National Academy of Sciences.

[12] Zhang F, Kurokawa K, Lassoued A, Crowell JA, Miller DT (2019). Cone photoreceptor classification in the living human eye from photostimulationinduced phase dynamics. Proc. Natl. Acad. Sci..

[13] Azimipour M, Migacz JV, Zawadzki RJ, Werner JS, Jonnal RS (2019). Functional retinal imaging using adaptive optics swept-source OCT at 1.6 MHz. Optica.

[14] Kennedy BF, Kennedy KM, Sampson DD (2014). A review of optical coherence elastography: Fundamentals, techniques and prospects. IEEE J. Sel. Top. Quantum Electron..

[15] Chin L (2014). Analysis of image formation in optical coherence elastography using a multiphysics approach. Biomed. Opt. Express.

[16] Wang RK, Kirkpatrick S, Hinds M (2007). Phase-sensitive optical coherence elastography for mapping tissue microstrains in real time. Appl. Phys. Lett..

[17] An L, Chao J, Johnstone M, Wang RK (2013). Noninvasive imaging of pulsatile movements of the optic nerve head in normal human subjects using phasesensitive spectral domain optical coherence tomography. Opt. Lett..

[18] Li P (2012). Phase-sensitive optical coherence tomography characterization of pulse-induced trabecular meshwork displacement in *ex vivo* nonhuman primate eyes. J. Biomed. Opt.

[19] Spahr H (2015). Imaging pulse wave propagation in human retinal vessels using full-field swept-source optical coherence tomography. Opt. Lett..

[20] van Brug, H. & Somers, P. A. A. M. Speckle decorrelation: Observed, explained, and tackled. In Jacquot, P. & Fournier, J.-M. (eds) *Interferometry in Speckle Light*, 11–18 (Springer Berlin Heidelberg, Berlin, Heidelberg, 2000).

[21] Knox KT, Thompson BJ (1974). Recovery of images from atmospherically degraded short-exposure photographs. Astrophys. J..

[22] Lohmann AW, Weigelt G, Wirnitzer B (1983). Speckle masking in astronomy: triple correlation theory and applications. Appl. Opt..

[23] Ayers GR, Northcott MJ, Dainty JC (1988). Knox–Thompson and triple-correlation imaging through atmospheric turbulence. J. Opt. Soc. Am. A.

[24] Lannes A (1989). Backprojection mechanisms in phase-closure imaging. Bispectral analysis of the phase-restoration process. Exp. Astron.

[25] Marron JC, Sanchez PP, Sullivan RC (1990). Unwrapping algorithm for least-squares phase recovery from the modulo 2π bispectrum phase. J. Opt. Soc. Am. A.

[26] Haniff CA (1991). Least-squares Fourier phase estimation from the modulo 2π bispectrum phase. J. Opt. Soc. Am. A.

[27] Roggemann, M., Welsh, B. & Hunt, B. *Imaging Through Turbulence. Laser & Optical Science & Technology* (Taylor & Francis, 1996).

[28] Mikurda K, Der Lühe OV (2006). High resolution solar speckle imaging with the extended Knox–Thompson algorithm. Sol. Phys..

[29] Bamler, R. & Just, D. Phase statistics and decorrelation in SAR interferograms. In Geoscience and Remote Sensing Symposium, 1993. IGARSS ’93. Better Understanding of Earth Environment., International, 980–984 vol.3, 10.1109/IGARSS.1993.322637 (1993).

[30] Zebker HA, Villasenor J (1992). Decorrelation in interferometric radar echoes. IEEE T. Geosci. Remote.

[31] Lanari, R. *et al*. An overview of the Small BAseline Subset algorithm: A DInSAR technique for surface deformation analysis. In Wolf, D. & Fernández, J. (eds) Deformation and Gravity Change: Indicators of Isostasy, Tectonics, Volcanism, and Climate Change, 637–661 (Birkhäuser Basel, Basel, 2007).

[32] Ferretti A, Prati C, Rocca F (2001). Permanent scatterers in SAR interferometry. IEEE T. Geosci. Remote.

[33] Sanderson C, Curtin R (2016). Armadillo: a template-based C++ library for linear algebra. J. Open Source Softw.

[34] Sanderson C, Curtin R (2018). A user-friendly hybrid sparse matrix class in C++. Lect. Notes Comput. Sci.

[35] Deledalle C, Denis L, Tupin F (2011). NL-InSAR: Nonlocal interferogram estimation. IEEE T. Geosci. Remote.

[36] Lucas A (2014). Insights into Titan’s geology and hydrology based on enhanced image processing of Cassini RADAR data. J. Geophys. Res. –Planet.

[37] Liba O (2017). Speckle-modulating optical coherence tomography in living mice and humans. Nat. Commun..

[38] Hillmann D (2016). Aberration-free volumetric high-speed imaging of *in vivo* retina. Sci. Rep.

