# Supplementary material for: Phase-sensitive interferometry of decorrelated speckle patterns
Source: Sci Rep. 2019 Aug 13;9:11748. doi: 10.1038/s41598-019-47979-8 (PMC6692410; doi:10.1038/s41598-019-47979-8)
Supplement: Supplementary file 1 — Supplementary information [file 41598_2019_47979_MOESM1_ESM.pdf]

# Phase-sensitive interferometry of decorrelated speckle patterns

## Supplementary information

**Hendrik Spahr**[1,2], **Clara Pfäffle**[1,2], **Sazan Burhan**[1], **Lisa Kutzner**[1,2], **Felix Hilge**[1,2], **Gereon Hüttmann**[1,2,3], **and Dierck Hillmann**[1,4,*]

[1]Institute of Biomedical Optics, University of Lübeck, Peter-Monnik-Weg 4, 23562 Lübeck, Germany
[2]Medical Laser Centre Lübeck GmbH, Peter-Monnik-Weg 4, 23562 Lübeck, Germany
[3]Airway Research Center North (ARCN), Member of the German Center for Lung Research (DZL), Germany
[4]Thorlabs GmbH, Maria-Goeppert-Straße 9, 23562 Lübeck, Germany
[*]dhillmann@thorlabs.com

## Contents

Additional Figures S1, S2, S3, and S4.

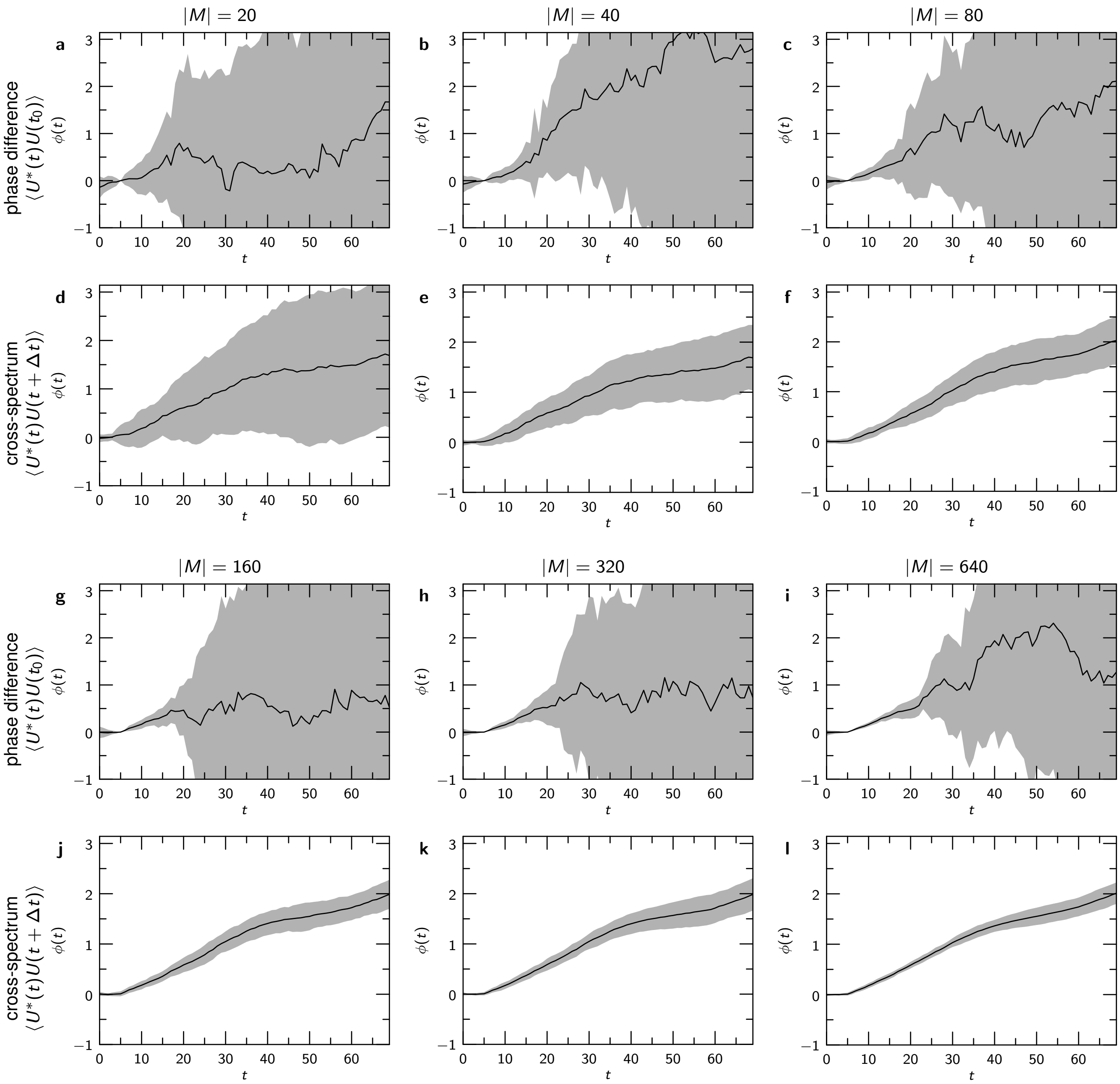


**Figure S1.** Simulation results (compare Fig. 4 in the main text) for various averaging parameters of sizes $|M|$ between $|M| = 20$ and $|M| = 640$ using both standard phase difference method (a-c and g-i) and the extended Knox-Thompson method (d-f and j-l). For all averaging sizes, the extended Knox-Thompson method shows reduced fluctuations and better reproduction of the input curves compared to phase differences. Neither method shows any significant bias in their estimation of the phase response.

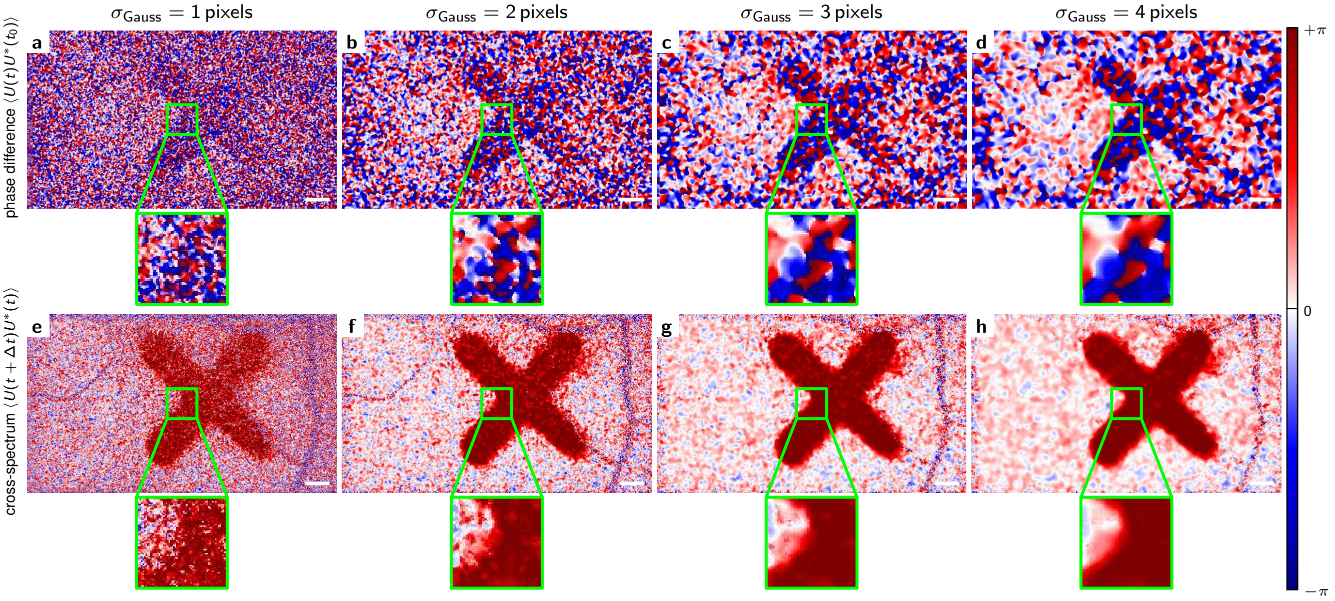


**Figure S2.** Experimental results (compare Fig. 5 in the main text) for various averaging parameters, here represented by the size of the applied Gaussian filter $\sigma_{\mathrm{Gauss}}$, using both standard phase difference method (a-d) and the extended Knox-Thompson method (e-h). For all filters, the extended Knox-Thompson method shows reduced fluctuations compared to phase differences.

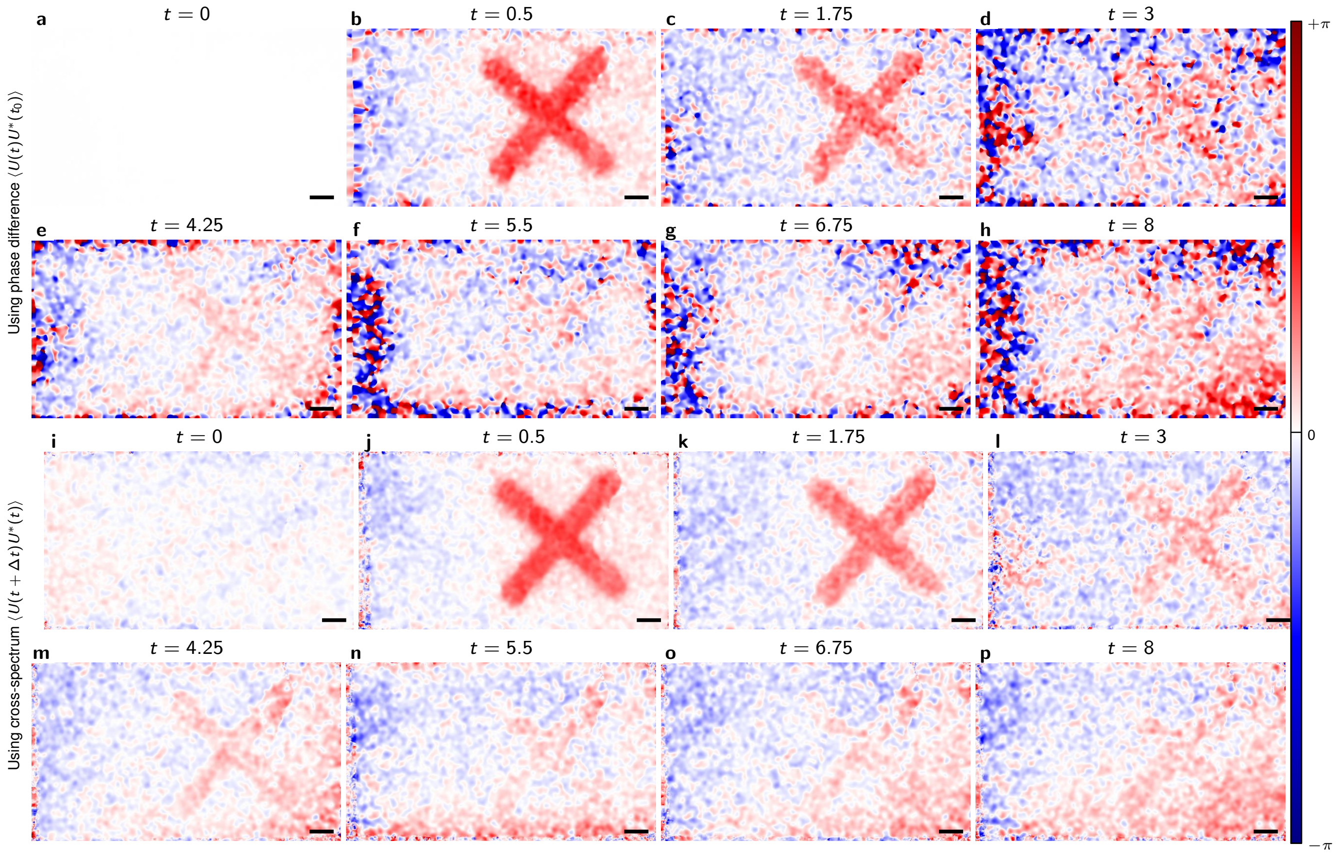


**Figure S3.** Experimental results (comparable to Fig. 5 in the main text), albeit only with a brief stimulation of 200 ms, showing the initial increase and the following partial relaxation of the signal over 8 s. In both methods a slight remaining 'x' is still visible at the end of the measurement indicating that there is remaining expansion in the stimulated area compared to the initial time. The extended Knox-Thompson method shows reduced noise, especially close to the edges at later times (compare f, g, and h with n, o, and p, respectively).

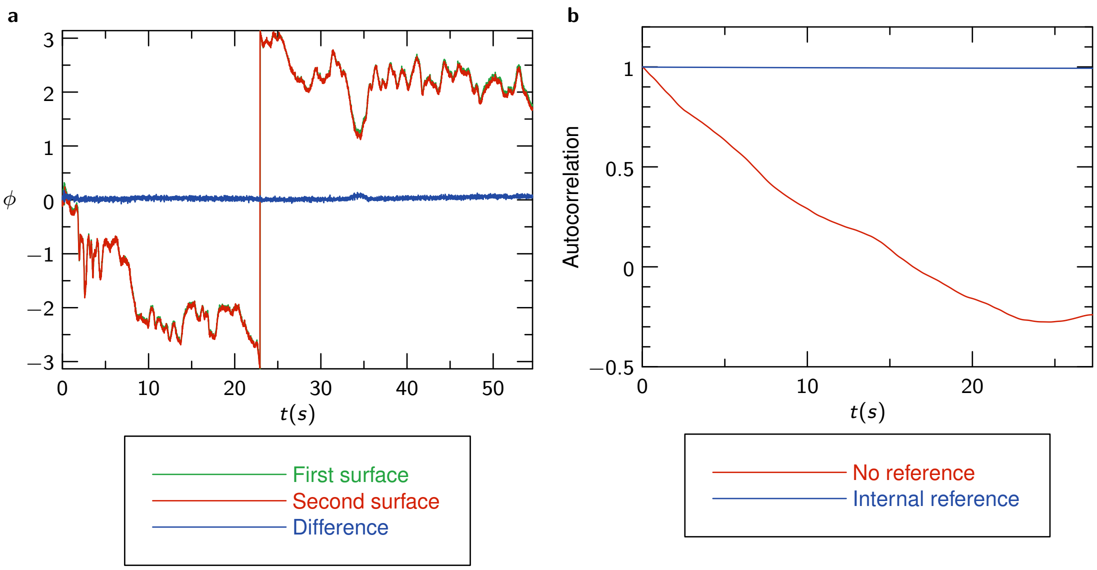


**Figure S4.** Phase stability of the OCT system used for this study. It was evaluated by measuring phases from a cover slip over more than 50 s and evaluation the autocorrelation over more than 25 s of the complex wave fields for certain depths. a) Phases from two layers of the cover slip (red and green) and their phase difference (blue) for one single lateral position of the coverslip. b) Autocorrelation of the complex field corresponding to one surfaces (red) and to the phase difference of both surfaces (blue). The autocorrelation is averaged over multiple lateral positions. The measurement demonstrates that phases referenced to a different layer from the same measurement can create phase stability exceeding 30 s.
